# Divergent successional responses and environmental drivers of soil bacterial and fungal communities in subalpine forests of the Eastern Qilian Mountains

**DOI:** 10.3389/fmicb.2026.1968766

**Published:** 2026-10-09

**Authors:** Zhengni Chen, Wenzhen Zong, Lei Ling, Quanlin Ma

**Affiliations:** 1 Gansu Academy of Forestry, Lanzhou, China; 2 Xinglong Mountain Forest Ecosystem Positioning Research Station, Lanzhou, China

**Keywords:** bacterial–fungal divergent responses, ectomycorrhizal fungi, environmental drivers, soil microbial community assembly, soil pH, subalpine forest succession

## Abstract

**Introduction:**

Soil microbial communities and extracellular enzyme activities play critical roles in regulating carbon (C), nitrogen (N), and phosphorus (P) cycling in forest ecosystems. However, whether bacterial and fungal communities exhibit similar or divergent successional trajectories, and how environmental factors regulate these responses during long-term subalpine forest succession, remain poorly understood.

**Methods:**

We investigated a natural successional sequence from broadleaved mixed forest to mature *Picea wilsonii* forest in Xinglong Mountain, Eastern Qilian Mountains, using high-throughput sequencing combined with measurements of soil physicochemical properties, microbial biomass, and extracellular enzyme activities.

**Results:**

Forest succession significantly altered soil environmental conditions, with surface soil pH increasing from 7.15 at the S1 stage to 7.49 at the S5 stage, whereas total phosphorus (TP) content decreased during early succession and subsequently remained relatively stable. Forest succession significantly influenced bacterial and fungal community composition but did not significantly affect α-diversity. Bacterial communities exhibited stage-specific shifts in the relative abundances of dominant taxa across the successional stages, whereas surface soil fungal communities showed more pronounced compositional reorganization. Mantel tests revealed that bacterial community composition was significantly associated with a broad suite of soil resource variables and environmental conditions, including pH, C and N availability, microbial biomass, and hydrolytic enzyme activities, whereas fungal community composition was primarily associated with soil pH and TP.

**Discussion:**

Collectively, our findings demonstrate that soil bacterial and fungal communities exhibit divergent successional response strategies during subalpine forest development in the Eastern Qilian Mountains. Bacterial communities were shaped by multiple resource-related and environmental factors, whereas fungal communities were more closely linked to soil pH and TP. This study reveals the divergent successional responses of soil bacterial and fungal communities during subalpine forest development under calcareous soil conditions, providing new insights into microbial community assembly processes and environmental regulation during forest succession.

## 1 Introduction

Forest succession drives the reorganization of soil microbial community composition and ecological functions by altering plant communities, carbon inputs, and soil environmental conditions (Bardgett and van der Putten, 2014; Walker and Wardle, 2014). As succession progresses, vegetation turnover and changes in resource inputs further regulate soil nutrient availability and microenvironmental conditions, thereby shaping microbially mediated carbon and nitrogen cycling processes (van der Putten et al., 2013; Cline and Zak, 2015). Soil microorganisms and their extracellular enzyme activities serve as key regulators of biogeochemical cycling, playing essential roles in maintaining forest ecosystem functioning and stability (Sinsabaugh et al., 2008; Schimel and Schaeffer, 2012). Therefore, understanding microbial community succession and the underlying environmental controls is critical for elucidating the mechanisms governing forest ecosystem development and recovery. However, microbial responses to forest succession are not universal, but instead vary depending on forest type, successional stage, and prevailing soil environmental conditions (Fierer et al., 2012; Tedersoo et al., 2014; Zhou and Ning, 2017). Although forest succession often promotes increases in microbial diversity and network complexity, different microbial groups may exhibit contrasting or even non-linear successional trajectories due to their distinct ecological strategies and resource acquisition mechanisms (Shade et al., 2012; He et al., 2022; Wang et al., 2025). Therefore, disentangling taxon-specific successional responses and their environmental determinants is essential for advancing our understanding of belowground community assembly and ecosystem functioning during forest development.

Bacteria and fungi, as the dominant constituents of soil microbial communities, differ fundamentally in their resource acquisition strategies, ecological functions, and environmental sensitivities, potentially resulting in contrasting successional trajectories (Peay et al., 2016; Zhou and Ning, 2017). Bacteria generally exhibit high metabolic versatility and substantial functional redundancy, with their community dynamics largely regulated by soil pH, carbon availability, carbon-to-nitrogen stoichiometry, and nutrient accessibility (Louca et al., 2018; Shang et al., 2021). In contrast, fungi, particularly ectomycorrhizal fungi, maintain closer ecological linkages with plant-derived carbon inputs and nutrient acquisition processes, rendering their community composition more responsive to shifts in vegetation composition and organic matter quality (Smith and Read, 2008; Phillips et al., 2013). Increasing evidence indicates that fungal communities often undergo more pronounced compositional turnover than bacterial communities during forest succession, reflecting distinct ecological strategies among microbial taxa in response to long-term environmental changes (Lin et al., 2021; Wang et al., 2025). However, the environmental mechanisms driving these divergent successional responses between bacterial and fungal communities remain insufficiently resolved.

Soil pH is a critical environmental filter governing microbial community composition and ecosystem functioning, exerting strong control over the distribution of bacterial and fungal communities at the global scale (Lauber et al., 2009; Rousk et al., 2010). In many coniferous forest ecosystems, tree species composition and litter characteristics can promote soil acidification by altering organic matter decomposition and nutrient cycling (Augusto et al., 1998; Finzi et al., 1998). However, pH trajectories during forest succession are not determined solely by vegetation change but are jointly regulated by parent material, initial soil pH, and soil buffering capacity (Jobbágy and Jackson, 2003; Walker and Wardle, 2014). In calcareous soils, carbonate buffering and mineral weathering can enhance soil resistance to acidification, thereby attenuating pH changes associated with vegetation development and organic matter decomposition (Lapenis et al., 2004). Previous evidence has shown that soil pH can increase along forest successional gradients in calcareous soils (Wei et al., 2012), contrasting with the acidification pattern frequently reported for coniferous forest ecosystems. This suggests that pedogenic conditions can modify soil pH trajectories during forest succession and, consequently, alter the environmental filtering and assembly of soil microbial communities. Nevertheless, how pH dynamics under calcareous soil conditions shape the divergent assembly trajectories of bacterial and fungal communities during forest succession remains poorly understood.

The subalpine *Picea wilsonii* forests in the Eastern Qilian Mountains encompass a natural successional sequence from broadleaved mixed forests to mature *P. wilsonii* forests under typical calcareous soil conditions, providing an ideal system for examining how forest succession regulates belowground microbial processes beyond the conventional acidification paradigm of coniferous ecosystems. In this study, we employed a space-for-time substitution approach and integrated soil physicochemical properties, microbial biomass, extracellular enzyme activities, and high-throughput sequencing to elucidate the shifts in soil environmental conditions, microbial community composition, and their underlying environmental drivers throughout forest succession. Furthermore, we compared the successional response strategies of bacterial and fungal communities across different developmental stages. Based on current ecological theories, we proposed the following hypotheses: (H1) Under calcareous soil conditions, forest succession modifies soil pH, nutrient availability, and resource supply, thereby driving directional changes in microbial community structure along the successional gradient; however, the magnitude of these responses differs among microbial groups due to their distinct ecological strategies. (H2) Bacterial and fungal communities exhibit contrasting successional response strategies: bacterial communities are primarily regulated by soil resource availability and environmental filtering and maintain structural stability through shifts in dominant taxa, whereas fungal communities, owing to their stronger dependence on plant-derived carbon inputs and mycorrhizal associations, exhibit greater compositional sensitivity to forest succession.

## 2 Materials and methods

### 2.1 Site description

This study was conducted in the Xinglong Mountain National Nature Reserve, Gansu Province, located in the Eastern Qilian Mountains, which represents a “green island” at the western margin of the Chinese Loess Plateau. The region experiences a typical warm temperate continental monsoon climate, with an average annual temperature of 6.5 °C and annual precipitation of 669 mm, of which 149.79 mm occurs from July to September. The annual sunshine duration is approximately 2008.6 h, corresponding to a sunshine percentage of about 54%. The predominant soil type is cinnamon soil, developed from limestone parent material, with an average soil depth of approximately 1.2 m. Forests are mainly distributed between 2,200 and 3,000 m above sea level and consist primarily of tree and shrub communities, with distinct vegetation zonation along slopes. Along shaded slopes, vegetation composition changes with increasing elevation, forming a successional sequence dominated by *Populus davidiana–Betula platyphylla* mixed forests, *B. platyphylla–Picea wilsonii* mixed forests, and mature *P. wilsonii* pure forests. *P. wilsonii* represents the late-successional dominant tree species and constitutes the climax community in this region.

Based on long-term permanent plot monitoring data and successional records, the forest chronosequence in Xinglong Mountain was categorized into five successional stages: *P. davidiana–B. platyphylla* mixed forest (S1), B. *platyphylla–P. wilsonii* mixed forests (S2–S3), middle-aged *P. wilsonii* forest (S4), and near-mature *P. wilsonii* forest (S5) (Figure 1). To improve the comparability among successional stages, all plots were selected from comparable slope aspects, parent materials, and soil types. The basic stand characteristics of each successional stage are summarized in Table 1.

**Figure 1:**
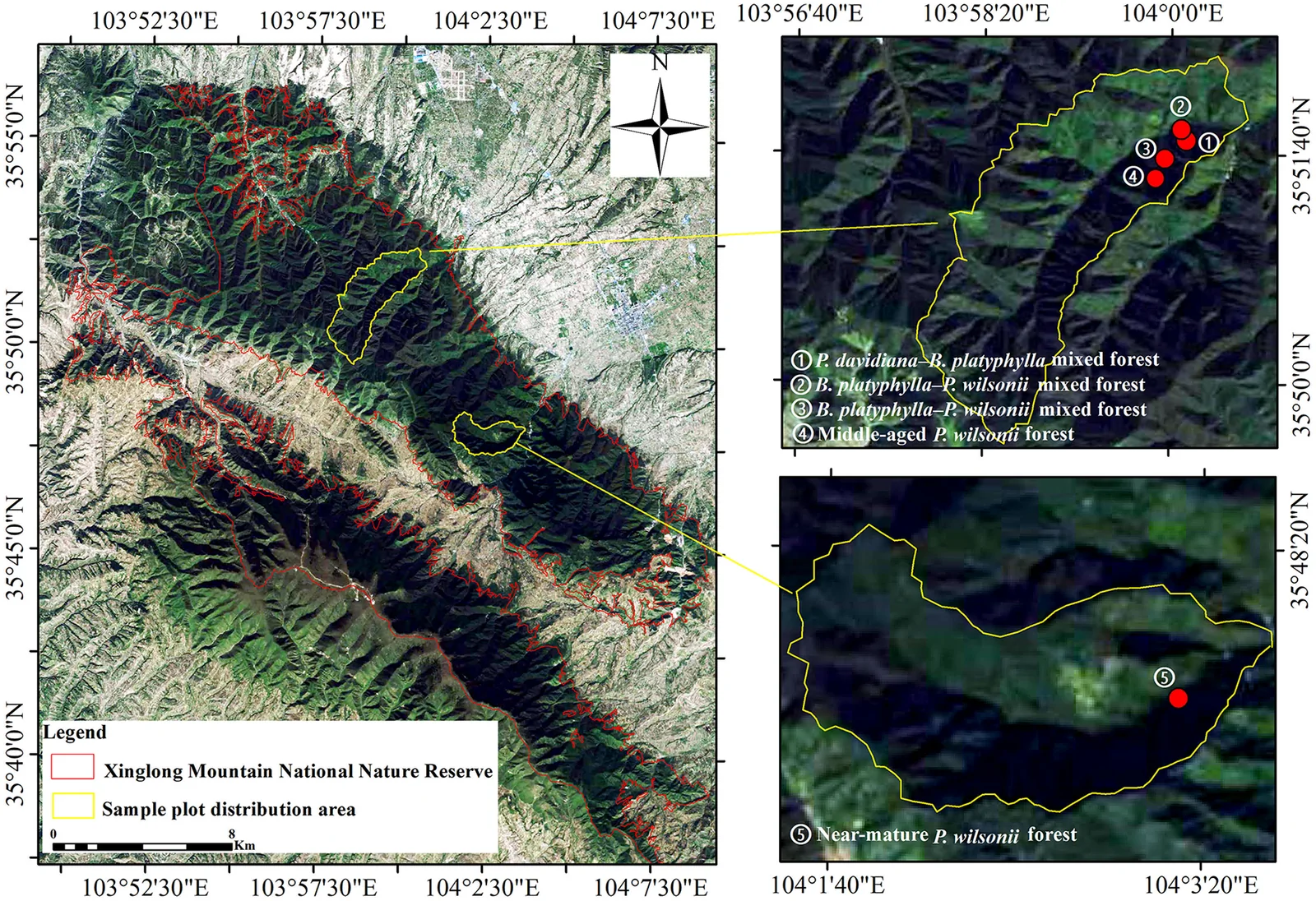
A map of the locality of the study area.

**Table 1:** Stand characteristics of forest communities across different succession stages in Xinglong Mountain.

Succession stage	Stand types	Dominant species	Mean DBH (cm)	Mean tree height (m)	Stand age (a)	Stand density (trees ha^−1^)	Canopy density	Altitude (m)
S1	Broadleaf mixed forest	*P. davidiana, B. platyphylla*	15.58	11.92	71	970	0.85	2,285
S2	Coniferous–broadleaf mixed forest	*B. platyphylla, P. wilsonii*	17.67	10.19	63	920	0.85	2,376
S3	Coniferous–broadleaf mixed forest	*B. platyphylla, P. wilsonii*	15.86	9.21	69	1325	0.9	2,393
S4	Middle-aged *P. wilsonii* forest	*P. wilsonii*	17.00	12.67	86	1286	0.9	2,406
S5	Near-mature *P. wilsonii* forest	*P. wilsonii*	36.47	22.09	145	377	0.65	2,440

### 2.2 Sampling design and field investigation

Field sampling was conducted during the growing season (16–17 July 2024). Soil samples from all five successional stages were collected within this 2-day window under consecutive sunny conditions to reduce the potential influence of short-term climatic fluctuations. The environmental conditions during the sampling period, including air temperature, relative humidity, soil temperature, and soil water content, showed relatively limited temporal variation and were used to describe the short-term environmental conditions of the study area at the time of soil sampling (Supplementary Table S1). Three representative plots (20 m × 20 m) were established for each successional stage, with a minimum distance of 50 m between adjacent plots. Within each plot, tree species composition, stand age, tree height, and diameter at breast height (DBH) were recorded. Canopy closure was visually estimated in the field. Soil samples were collected from the 0 to 20 and 20 to 40 cm soil layers in the inner portion of each plot to minimize potential edge effects. For each soil depth, three soil cores were collected within each plot and thoroughly homogenized to form one composite sample per plot. Thus, the three plots within each successional stage constituted three independent plot-level replicates for each soil depth. Immediately after collection, the soil was passed through a 2-mm sieve in the field to remove visible plant residues and gravel. The homogenized samples were transported to the laboratory under refrigerated conditions. A portion of the fresh soil was immediately stored at −80 °C for subsequent DNA extraction, while another portion was stored at 4 °C for analyses of soil microbial biomass, Dissolved Organic Carbon (DOC), Dissolved Organic Nitrogen (DON), and extracellular enzyme activities. The remaining samples were air-dried and used to determine Total Carbon (TC), Total Nitrogen (TN), Total Phosphorus (TP), Soil Organic Carbon (SOC) and soil pH.

### 2.3 Determination of soil physicochemical and biological properties

Soil pH was determined using the potentiometric method with a soil-to-water ratio of 2.5:1 (w/v). Soil TC and TN were measured using an elemental analyzer (Elementar, Langenselbold, Germany). Soil TP was determined using the molybdenum blue colorimetric method following digestion with an H_2_SO4–HClO4 mixture. SOC was quantified using the potassium dichromate oxidation method (Nelson and Sommers, 1982; Bao, 2000). DOC was extracted with deionized water and quantified using a TOC/TN analyzer (Multi N/C 2100, Analytik Jena, Germany).

DON was calculated as the difference between total dissolved nitrogen and inorganic nitrogen. Total dissolved nitrogen was determined using the persulfate oxidation method, whereas NH4?-N and NO3?-N concentrations were measured using the modified indophenol blue method and the Griess reagent method, respectively (Doyle et al., 2004; Hood-Nowotny et al., 2010). Soil microbial biomass carbon, nitrogen and phosphorus (MBC, MBN and MBP) were determined using the chloroform fumigation–K_2_SO4 extraction method (Vance et al., 1987). The activities of extracellular hydrolytic enzymes associated with carbon, nitrogen, and phosphorus acquisition, including β-glucosidase (BG), *N*-acetyl-β-D-glucosaminidase (NAG), Leucine Aminopeptidase (LAP), and Acid Phosphatase (ACP), were determined using a fluorometric microplate assay with fluorescent substrates (Saiya-Cork et al., 2002; DeForest, 2009). The activities of oxidative enzymes, including phenol oxidase (PPO) and peroxidase (POD), were measured using L-DOPA as the substrate through a microplate colorimetric assay (Saiya-Cork et al., 2002).

### 2.4 DNA extraction, PCR amplification, and Illumina sequencing

Soil bacterial and fungal community compositions were characterized using high-throughput amplicon sequencing. Total genomic DNA was extracted from approximately 0.5 g of fresh soil using the MN NucleoSpin 96 Soil Kit (Macherey–Nagel, Germany). The concentration and integrity of extracted DNA were evaluated using a NanoDrop 2,000 spectrophotometer and 1% agarose gel electrophoresis, respectively. Bacterial communities were characterized by amplifying the V3–4 hypervariable regions of the 16S rRNA gene using the primer pair 341F (5′-CCTACGGGNGGCWGCAG-3′) and 805R (5′-GACTACHVGGGTATCTAATCC-3′) (Herlemann et al., 2011). Fungal communities were characterized by amplifying the ITS2 region using the primer pair fITS7F (5′-GTGARTCATCGAATCTTTG-3′) and ITS4R (5′-TCCTCCGCTTATTGATATGC-3′) (Ihrmark et al., 2012). The PCR products were purified, quantified, and pooled at equimolar concentrations to construct sequencing libraries, which were subsequently sequenced on an Illumina NovaSeq 6,000 platform using paired-end sequencing technology. Raw sequencing reads were processed using the QIIME2 pipeline for quality control and preprocessing (Bolyen et al., 2019). This process included the removal of low-quality reads, sequences containing ambiguous bases (N), primer mismatches, and chimeric sequences. The DADA2 plugin was subsequently applied for sequence denoising, paired-end read merging, and chimera filtering, generating high-resolution amplicon sequence variants (ASVs) (Callahan et al., 2016). Taxonomic classification of bacterial and fungal ASV representative sequences was performed against the SILVA database (v138) and the UNITE database, respectively. To minimize the potential influence of sequencing depth variation among samples, ASV tables were rarefied to the lowest sequencing depth across all samples prior to downstream community analyses.

### 2.5 Statistical analyses

All statistical analyses and graphical visualizations were conducted in R software (version 4.5). Data distribution normality was first assessed using the Kolmogorov–Smirnov test, and variables deviating from normality were appropriately transformed before subsequent analyses. One-way analysis of variance (ANOVA) was used to evaluate the effects of successional stage on soil physicochemical properties, microbial biomass, extracellular enzyme activities, and microbial α-diversity indices, followed by Tukey's HSD test for pairwise comparisons among successional stages. Microbial community structure analyses were performed based on ASV abundance matrices. The ASV abundance tables were rarefied to the minimum sequencing depth and Hellinger-transformed prior to community analyses. The microeco package was used to characterize microbial taxonomic composition and identify dominant bacterial and fungal taxa. The relative abundances of dominant family were standardized using Z-score transformation, and hierarchical clustering analysis was conducted to generate heatmaps illustrating shifts in microbial community composition across successional stages. Linear mixed-effects models were employed to assess the effects of successional stage, soil depth, and their interaction on bacterial and fungal α-diversity indices, including Richness, Shannon diversity, and Pielou's evenness. In these models, successional stage, soil depth, and their interaction were treated as fixed effects, whereas plot identity was included as a random intercept to account for spatial dependence among samples. Microbial α-diversity indices, including richness, Shannon diversity, and Simpson diversity, were calculated from bacterial and fungal ASV abundance matrices using the vegan (version 2.7.2) and picante packages in R. Richness was defined as the observed number of ASVs per sample. Based on Bray–Curtis dissimilarity matrices, non-metric multidimensional scaling (NMDS) was performed to visualize differences in bacterial and fungal community structure among successional stages. The effects of successional stage and soil depth on microbial community structure were further tested using permutational multivariate analysis of variance (PERMANOVA; 999 permutations) implemented with the adonis function in the vegan package. Redundancy analysis (RDA) was conducted to evaluate the relationships between environmental variables and microbial community composition. Environmental variables significantly correlated with microbial community variation were identified using Pearson correlation analyses (*P* < 0.05). Partial Mantel tests (999 permutations), implemented in the linkET package (version 0.1.0), were subsequently performed to identify the key environmental factors driving variations in soil bacterial and fungal community structures.

## 3 Results

### 3.1 Changes in soil physicochemical, biological properties, and enzyme activities

Several soil physicochemical properties, microbial biomass, and extracellular enzyme activities varied significantly across successional stages and soil depths (Table 2). In the surface soil (0–20 cm), both soil pH and TP exhibited significant successional shifts. Specifically, soil pH increased progressively from 7.15 at the S1 stage to 7.49 at the S5 stage. In contrast, TP declined significantly during the early stages of succession and subsequently remained relatively stable in later stages. Variations in TC, TN, and SOC were primarily detected in the subsurface soil, where all three variables displayed a consistent decreasing–increasing pattern along the successional gradient, reaching their minimum values at the S4 stage (*P* < 0.05). DON showed an opposite unimodal pattern, increasing during the early successional stages and declining thereafter, with the highest concentration observed at the S4 stage (*P* < 0.05). Despite significant successional changes in several soil nutrient variables, MBC, MBN, and MBP remained relatively stable and did not differ significantly among successional stages. Extracellular enzyme activities exhibited distinct successional responses. The activity of BG showed a decreasing–increasing trend along the successional gradient and was significantly influenced by succession in the subsurface soil (*P* < 0.01), whereas the activities of NAG and ACP showed no significant variation among successional stages. Oxidative enzyme activities exhibited stronger responses to forest succession, with POD and PPO displaying significant unimodal patterns, characterized by an initial increase followed by a decline, in the subsurface and surface soils, respectively (*P* < 0.05).

**Table 2:** Soil physicochemical properties, microbial biomass, and enzyme activities across different succession stages in Xinglong Mountain.

Variable	Depth	S1	S2	S3	S4	S5	*P* value
pH	top	7.15 ± 0.10B	7.24 ± 0.09AB	7.19 ± 0.14B	7.34 ± 0.10AB	7.49 ± 0.05A	0.013
	sub	7.39 ± 0.31a	7.33 ± 0.12a	7.28 ± 0.07a	7.48 ± 0.22a	7.52 ± 0.05a	0.505
TC (%)	top	9.53 ± 1.13A	9.87 ± 1.80A	8.16 ± 3.24A	6.40 ± 1.29A	6.96 ± 1.66A	0.206
	sub	6.10 ± 1.01a	4.67 ± 1.08ab	3.50 ± 1.24ab	3.01 ± 0.68b	4.49 ± 0.73ab	0.024
TN (%)	top	0.75 ± 0.07A	0.76 ± 0.16A	0.58 ± 0.21A	0.47 ± 0.08A	0.50 ± 0.12A	0.074
	sub	0.50 ± 0.07a	0.37 ± 0.09ab	0.27 ± 0.07b	0.25 ± 0.04b	0.34 ± 0.06ab	0.007
TP (%)	top	1.04 ± 0.06A	0.85 ± 0.07B	0.71 ± 0.08B	0.71 ± 0.04B	0.80 ± 0.07B	< 0.001
	sub	1.01 ± 0.06a	0.61 ± 0.07bc	0.45 ± 0.09c	0.57 ± 0.06bc	0.70 ± 0.09b	< 0.001
SOC (mg/kg)	top	90.24 ± 11.43A	87.69 ± 7.46A	65.52 ± 11.61A	59.56 ± 10.32A	64.76 ± 17.92A	0.032
	sub	58.02 ± 11.12a	46.44 ± 9.85ab	32.76 ± 11.55ab	28.63 ± 6.05b	41.16 ± 8.17ab	0.026
DOC (mg/kg)	top	91.60 ± 4.06B	134.10 ± 7.92A	109.95 ± 20.48AB	141.05 ± 14.63A	113.91 ± 7.99AB	0.004
	sub	76.23 ± 3.88a	73.19 ± 20.30a	68.22 ± 15.09a	92.22 ± 6.92a	84.08 ± 6.33a	0.204
DON (mg/kg)	top	3.66 ± 0.74C	10.99 ± 1.77B	11.78 ± 0.79B	16.15 ± 2.34A	11.99 ± 1.39B	< 0.001
	sub	3.13 ± 0.54c	4.04 ± 0.46bc	5.20 ± 1.58bc	9.74 ± 0.88a	6.57 ± 1.37b	< 0.001
MBC (mg/kg)	top	469.29 ± 51.99A	493.49 ± 25.45A	466.14 ± 32.83A	420.77 ± 67.89A	431.09 ± 44.88A	0.368
	sub	214.91 ± 17.20a	212.30 ± 95.92a	190.70 ± 61.10a	177.89 ± 35.27a	219.12 ± 39.44a	0.873
MBN (mg/kg)	top	46.84 ± 7.04A	53.41 ± 6.29A	48.35 ± 5.76A	42.63 ± 8.23A	40.82 ± 5.65A	0.234
	sub	23.46 ± 2.20a	23.67 ± 11.20a	21.97 ± 7.51a	23.37 ± 3.39a	25.18 ± 4.10a	0.983
MBP (mg/kg)	top	17.45 ± 3.84A	17.01 ± 1.35A	17.72 ± 2.60A	15.57 ± 3.09A	15.20 ± 1.90A	0.717
	sub	7.07 ± 0.13a	6.71 ± 1.41a	5.94 ± 2.56a	6.47 ± 1.28a	5.96 ± 0.93a	0.857
BG (μmol/h/g)	top	58.16 ± 16.60A	57.55 ± 10.68A	50.10 ± 13.00A	29.92 ± 2.51A	43.07 ± 9.75A	0.066
	sub	34.89 ± 3.21a	32.71 ± 9.78ab	19.55 ± 5.26bc	16.13 ± 2.95c	23.54 ± 3.18abc	0.007
NAG (μmol/h/g)	top	19.28 ± 2.43A	25.38 ± 4.28A	27.78 ± 5.79A	22.06 ± 10.73A	17.75 ± 6.76A	0.374
	sub	9.07 ± 3.21a	12.56 ± 2.72a	14.00 ± 3.52a	10.12 ± 3.42a	8.64 ± 1.06a	0.192
ACP (μmol/h/g)	top	15.44 ± 1.13A	20.50 ± 8.15A	18.05 ± 5.53A	12.43 ± 0.34A	10.52 ± 2.02A	0.119
	sub	13.00 ± 0.28a	13.12 ± 5.33a	9.21 ± 1.91a	7.18 ± 3.10a	7.02 ± 0.42a	0.060
LAP (μmol/h/g)	top	37.26 ± 3.66A	21.27 ± 3.79A	15.39 ± 4.28A	37.29 ± 16.99A	21.64 ± 9.61A	0.047
	sub	18.88 ± 4.68a	15.56 ± 3.96a	10.87 ± 2.21a	14.50 ± 4.53a	19.10 ± 6.75a	0.248
POD (nmol/h/g)	top	722.47 ± 229.40A	1187.30 ± 100.00A	884.62 ± 179.94A	861.20 ± 162.27A	798.14 ± 176.50A	0.067
	sub	816.15 ± 224.75b	1097.21 ± 127.45ab	1448.54 ± 40.81a	931.46 ± 241.26b	790.93 ± 157.80b	0.005
PPO (nmol/h/g)	top	390.96 ± 148.84B	837.77 ± 319.72AB	1129.64 ± 58.46A	998.12 ± 241.38A	699.05 ± 237.23AB	0.017
	sub	778.32 ± 219.35a	1111.63 ± 50.22a	830.57 ± 188.81a	738.68 ± 153.77a	699.05 ± 112.38a	0.058

S1–5 represent the different successional stages; top indicates surface soil (0–20 cm), and sub indicates subsurface soil (20–40 cm). Different uppercase letters indicate significant differences among successional stages in surface soil (*P* < 0.05), whereas different lowercase letters indicate significant differences among successional stages in subsurface soil (*P* < 0.05). TC, total carbon; TN, total nitrogen; TP, total phosphorus; SOC, soil organic carbon; DOC, dissolved organic carbon; DON, dissolved organic nitrogen; MBC, microbial biomass carbon; MBN, microbial biomass nitrogen; MBP, microbial biomass phosphorus; BG, β-glucosidase; NAG, *N*-acetyl-β-D-glucosaminidase; ACP, acid phosphatase; LAP, leucine aminopeptidase; POD, peroxidase; PPO, polyphenol oxidase.

### 3.2 Taxonomic composition of soil bacterial and fungal communities

The soil bacterial community was primarily composed of *Acidobacteriota* (19.35%), *Proteobacteria* (19.16%), *Actinobacteriota* (16.52%), *Planctomycetota* (12.84%), and *Chloroflexi* (10.42%) (Figures 2A, B). Across the successional gradient, the relative abundance of *Actinobacteriota* in surface soil showed an overall increasing trend and reached its maximum at the S5 stage. In contrast, the relative abundance of *Planctomycetota* increased during succession and peaked at the S4 stage before declining, whereas *Chloroflexi* exhibited a continuous decreasing trend. In subsurface soil, the relative abundance of *Actinobacteriota* increased significantly during the early successional stages, reaching its highest value at the S2 stage, followed by a gradual decline in later stages. Conversely, *Planctomycetot*a displayed a decreasing–increasing pattern along the successional gradient. The relative abundance of *Gemmatimonadota* was consistently higher in surface soil than in subsurface soil. The fungal community was dominated by Basidiomycota (74.20%), Ascomycota (17.11%), and Zygomycota (6.01%) (Figures 2C, D). Basidiomycota represented the predominant fungal phylum across all successional stages and soil depths, accounting for the largest proportion of the fungal community. In surface soil, the relative abundance of Basidiomycota exhibited a unimodal response along the successional gradient, increasing during the early stages and declining in later stages, whereas Ascomycota showed an opposite pattern, with an initial decrease followed by an increase. In contrast, fungal community composition in subsurface soil remained relatively stable throughout succession, characterized by a consistently high dominance of Basidiomycota. The corresponding phylum-level relative abundances of individual plot-level replicates are presented in Supplementary Figure S1, allowing variation among replicates within each successional stage to be assessed.

**Figure 2:**
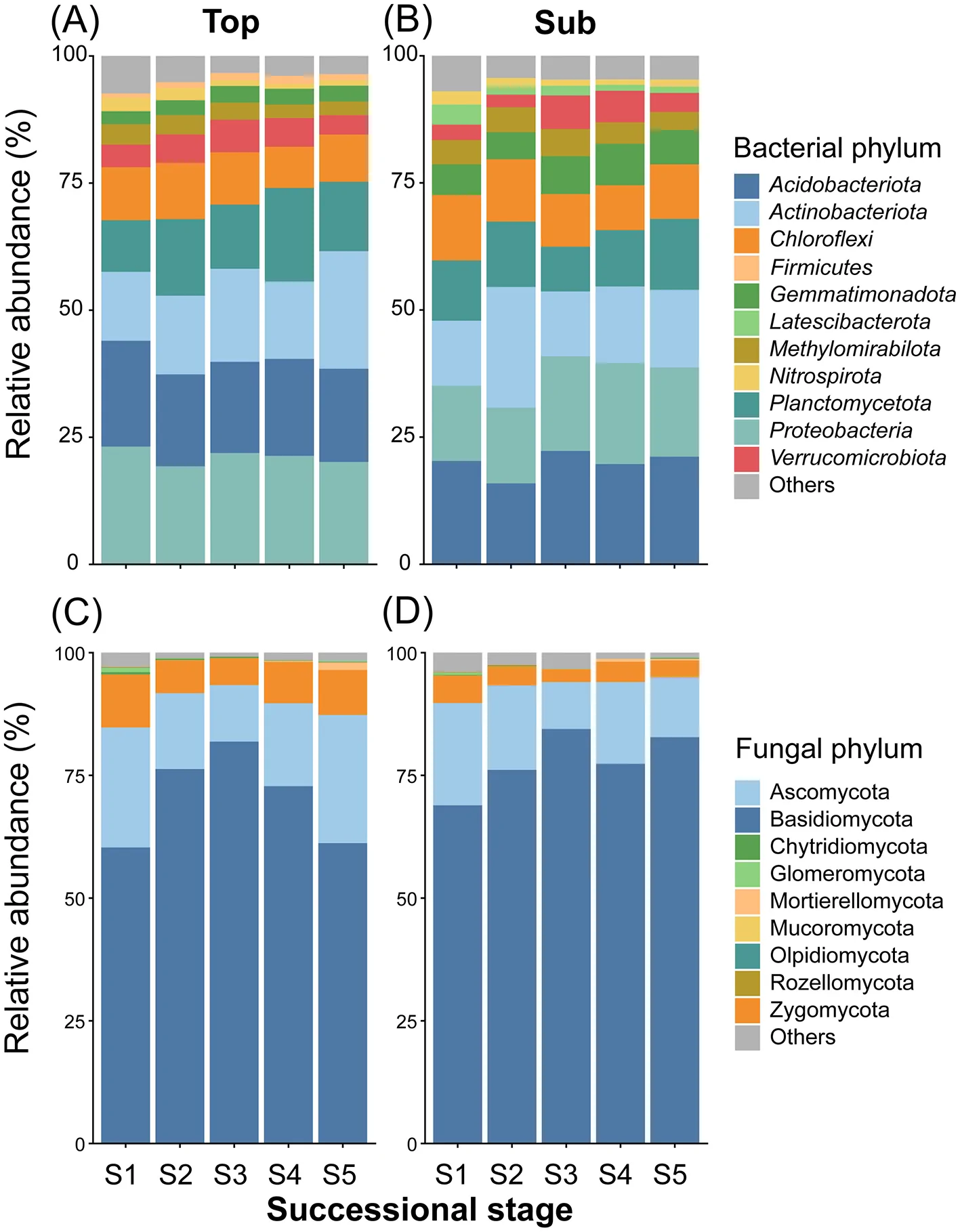
Relative abundances of soil bacterial **(A, B)** and fungal **(C, D)** phyla across different successional stages. S1–S5 represent different successional stages.

At the family level, bacterial and fungal communities exhibited distinct patterns of variation in the relative abundances of individual taxa across the successional stages. Bacterial communities were primarily characterized by the relatively high abundances of *Nitrosomonadacea, Pyrinomonadaceae, Pirellulaceae, Vicinamibacteraceae*, and *Gemmataceae* (Figure 3A; Supplementary Table S2). In surface soil, the relative abundance of *Nitrosomonadaceae* decreased from 3.12% at S1 to 0.95% at S4 and increased to 1.36% at S5, whereas that of *Nitrospiraceae* declined from 2.59 to 2.40% at S1 and S2, respectively, to 1.09% at S5. In contrast, *Vicinamibacteraceae* increased from 3.18% at S1to 3.82% at S5, *Micromonosporaceae* increased from 0.58% at S1 to 1.50% at S5. *Phyllobacteriaceae* differed significantly among successional stages in both surface and subsurface soils, with its relative abundance increasing from 0.37% at S1 to 0.88% at S4 in surface soil and from 0.16% to 0.59% in subsurface soil. The relative abundance of *Pirellulaceae* in surface soil also increased from 3.55% at S1 to 6.18% at S4. Soil bacterial communities primarily exhibited stage-specific shifts in the relative abundances of dominant taxa along the forest succession gradient.

**Figure 3:**
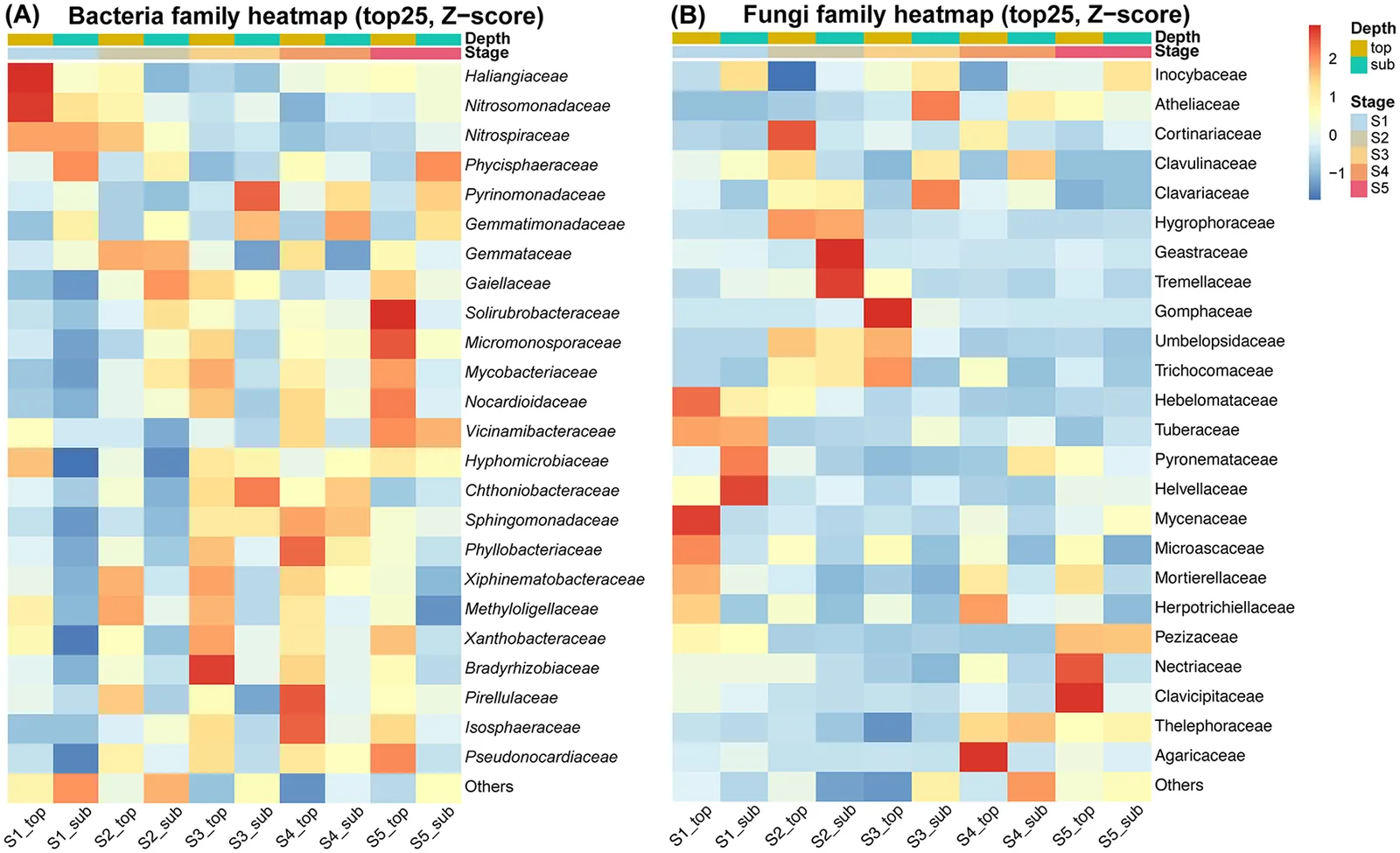
Heatmaps of the relative abundances of dominant bacterial **(A)** and fungal **(B)** family across forest successional stages. The heatmaps display the 25 most abundant family within each microbial group, with relative abundances standardized as Z-scores. The x-axis represents the forest successional stages (S1–S5) and soil depths (top and sub). The color gradient from blue to red indicates increasing relative abundance. The dendrogram on the left depicts the similarity in relative-abundance patterns among family-level taxa across the different groups. Labels containing “unclassified,” as well as genus-level taxa denoted by numbers, letters, or alphanumeric combinations, represent taxa that could not be further resolved to a formally recognized genus.

Fungal communities showed distinct variation in the relative abundances of individual families across successional stages, particularly in surface soils (Figure 3B; Supplementary Table S3). Inocybaceae was the most abundant fungal family, with mean relative abundances of 22.04 and 35.19% in surface and subsurface soils, respectively, followed by Mortierellaceae, with corresponding mean relative abundances of 6.81 and 3.20%. Although these two dominant families maintained relatively high relative abundances across both soil depths, their relative abundances did not differ significantly among successional stages, whereas several fungal taxa exhibited pronounced stage-specific shifts. Differences among successional stages were mainly observed in surface soil. The relative abundance of Atheliaceae increased from 0 at S1 to 0.59% at S5, whereas Hebelomataceae and Tuberaceae both showed their highest relative abundances at S1 (2.02% and 0.99%, respectively) and subsequently occurred at lower abundances. Hygrophoraceae reached its highest relative abundance at S2 (5.63%) and subsequently decreased to 0.01%. Clavariaceae and Clavicipitaceae also differed significantly among successional stages in surface soil. In subsurface soil, significant differences among successional stages were detected only for Atheliaceae, Hebelomataceae, and Helvellaceae.

### 3.3 Changes in soil microbial α-diversity

Although bacterial and fungal α-diversity indices exhibited some variations across successional stages and soil depths, neither successional stage, soil depth, nor their interaction significantly affected Richness, Shannon index, or Pielou's evenness (Figures 4A–F; Supplementary Table S4). Along the successional gradient from S1 to S5, bacterial Richness, Shannon index, and Pielou's evenness remained relatively stable, with no significant differences detected among successional stages or between soil depths (Figures 4A–C). Similarly, fungal Richness, Shannon index, and Pielou's evenness showed no significant responses to either successional stage or soil depth (Figures 4D–F; Supplementary Table S4). These findings suggest that, despite pronounced shifts in community composition, the overall richness, diversity, and evenness of soil microbial communities remained relatively stable during forest succession in the studied subalpine ecosystem.

**Figure 4:**
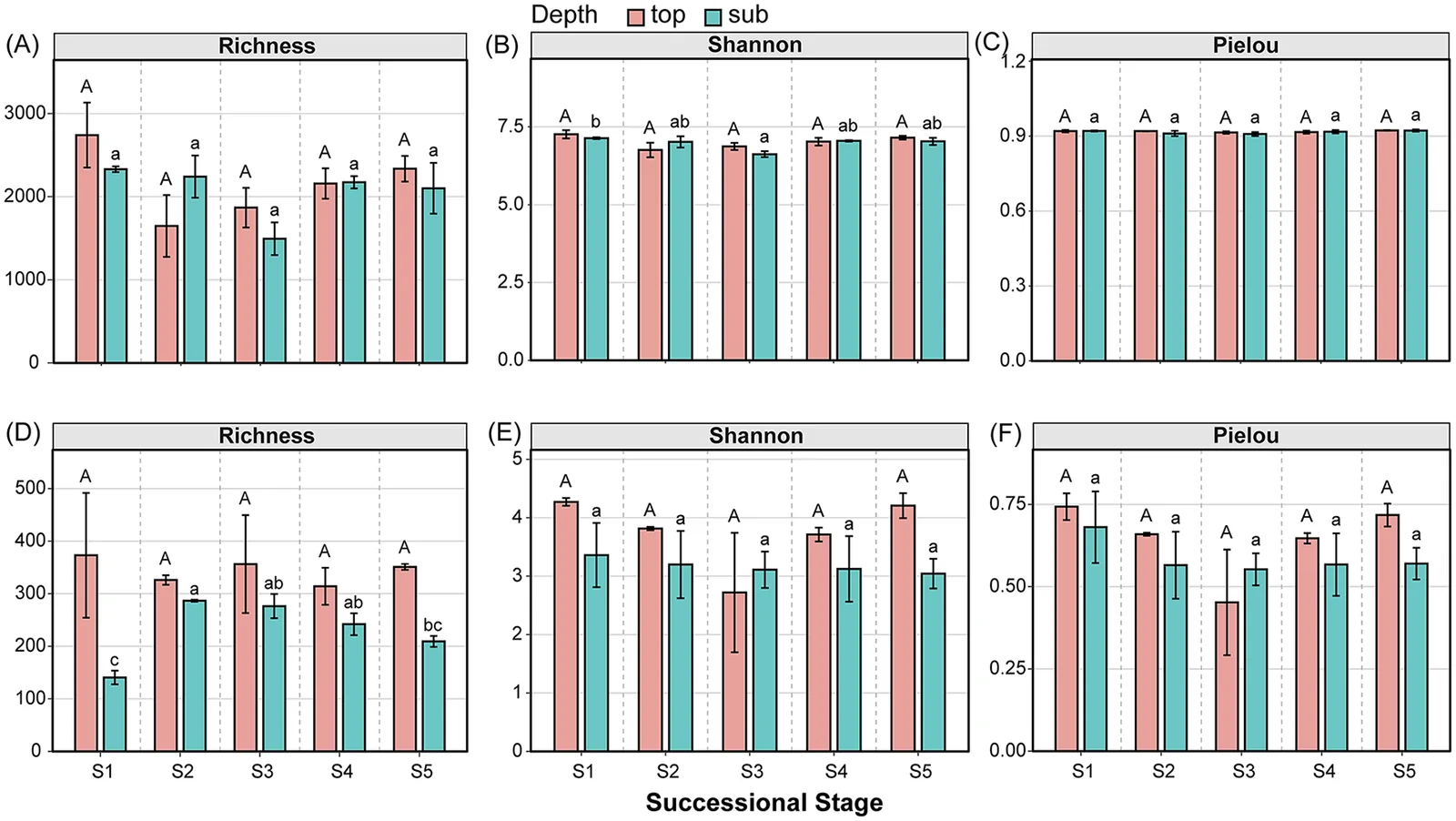
α-diversity indices (Richness, Shannon index, and Pielou's evenness) of soil fungal **(A–C)** and bacterial **(D–F)** communities across different successional stages and soil depths. Bars represent means ± standard errors. S1–S5 indicate different successional stages. Different uppercase letters indicate significant differences among successional stages in surface soil, whereas different lowercase letters indicate significant differences among successional stages in subsurface soil. Richness is defined as the observed number of ASVs per sample, while the Shannon diversity and Pielou's evenness indices are dimensionless.

### 3.4 Differentiation of soil microbial community structure

NMDS ordination revealed distinct successional shifts in both bacterial and fungal community structure along the NMDS1 axis (Figure 5). Bacterial and fungal communities at the S1 stage were primarily distributed on the negative side of NMDS1, whereas those at the S2 stage occupied an intermediate transitional position. In contrast, communities from the S3–5 stages were generally clustered on the positive side of NMDS1, indicating a directional and continuous reorganization of soil microbial community structure throughout forest succession. PERMANOVA further demonstrated that successional stage exerted significant effects on both bacterial and fungal community structures (Figure 5). For bacterial communities, successional stage explained 26.7% of the variation in community structure (R^2^_stage_ = 0.267, *P* < 0.01), whereas soil depth accounted for only 3.3% of the variation (R^2^_depth_ = 0.033) and showed no significant effect. Similarly, for fungal communities, succession stage explained 28.9% of the compositional variation (R^2^_stage_ = 0.289, *P* < 0.01), while soil depth explained 4.8% (R^2^_depth_ = 0.048, *P* < 0.05). Collectively, these results indicate that forest succession was the dominant ecological process driving the reorganization of soil bacterial and fungal communities, with substantially greater explanatory power than soil depth. Furthermore, fungal communities exhibited stronger responses to both successional progression and vertical soil heterogeneity than bacterial communities, suggesting that fungi may be more sensitive to environmental changes associated with forest development and vegetation transition.

**Figure 5:**
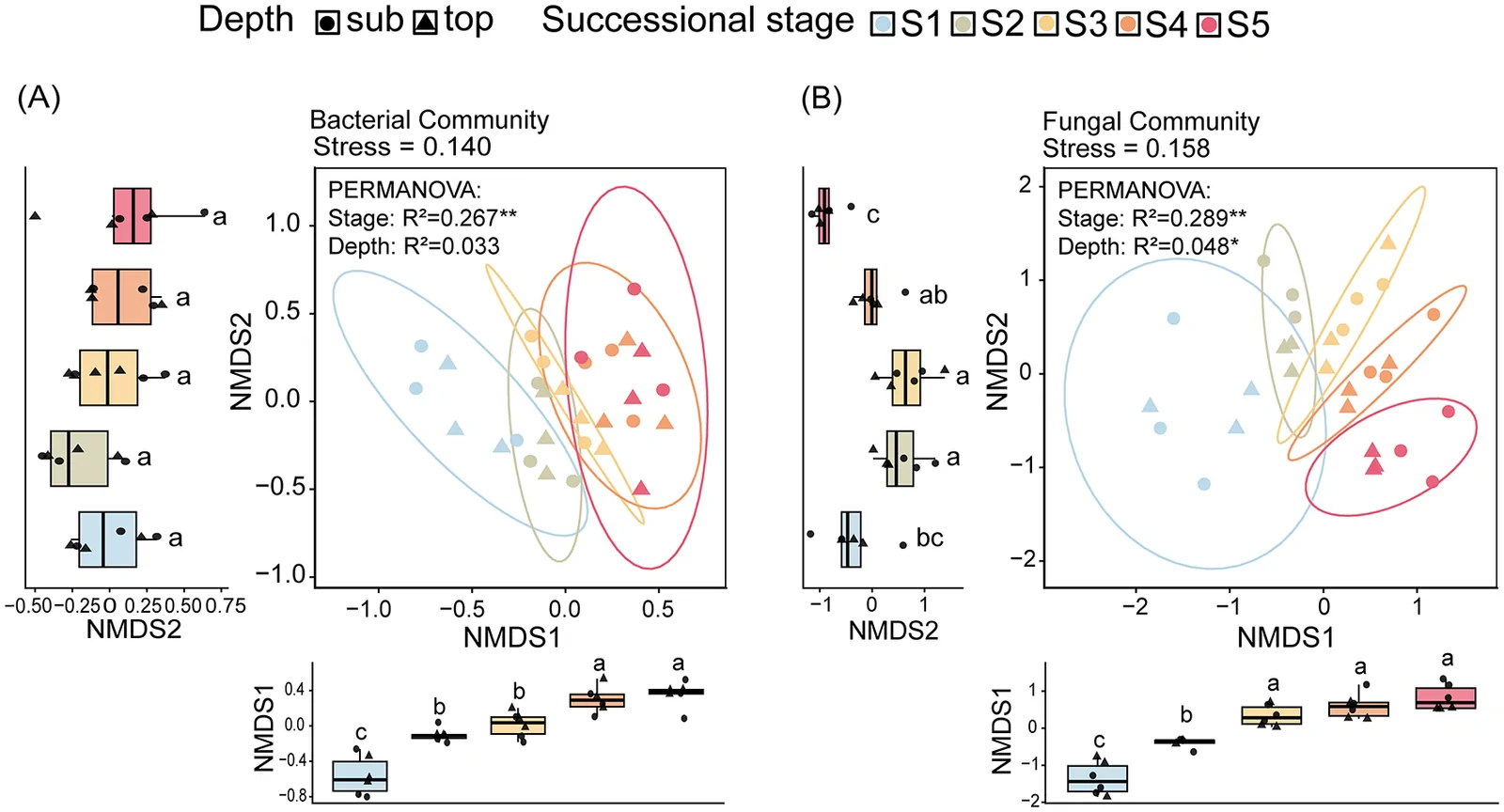
Non-metric multidimensional scaling (NMDS) ordination of bacterial **(A)** and fungal **(B)** community structure based on Bray–Curtis dissimilarity matrices. S1–S5 denote the different forest successional stages. The values following “Stage” and “Depth” represent R^2^ values, indicating the proportions of variation in community structure explained by forest succession and soil depth, respectively, as determined by PERMANOVA. * indicates *P* < 0.05, and ** indicates *P* < 0.01.

### 3.5 Major environmental drivers shaping soil microbial community composition

RDA revealed that variations in soil nutrient availability, microbial biomass, extracellular enzyme activities, and pH jointly contributed to the differentiation of bacterial and fungal community structures (Figure 6). For bacterial communities, the first two RDA axes explained 65.9% of the total variation, with RDA1 and RDA2 accounting for 35.0 and 30.9%, respectively (Figure 6A). SOC, TC, TN, MBC, MBN, and MBP were primarily associated with the negative axis of RDA1, whereas soil pH, NAG, LAP, BG, and ACP were positively correlated with RDA1. This distribution pattern indicates that bacterial community variation was closely linked to gradients of soil nutrient availability, microbial biomass, extracellular enzyme activities and pH. *Pirellulaceae, Xiphinematobacteraceae*, and *Bradyrhizobiaceae* exhibited distribution patterns consistent with SOC, TC, TN, and microbial biomass indicators, whereas *Pyrinomonadaceae* showed a strong association with pH. *Hthoniobacteraceae, Sphingomonadaceae*, and *Pyrinomonadaceae* were positioned on the positive direction of RDA1, *Nitrospiraceae* was positioned on the negative side of RDA1. For fungal communities, the first two RDA axes explained 75.9% of the total variation, with RDA1 and RDA2 accounting for 44.7 and 31.2%, respectively (Figure 6B). Similar to bacterial communities, SOC, TC, TN, MBC, MBN, MBP, PPO, BG, and LAP were primarily distributed along the negative direction of RDA1, whereas pH, TP, ACP, and NAG showed a positive association with RDA1. These patterns suggest that fungal community composition was also strongly related to gradients of soil nutrient, microbial biomass, extracellular enzyme activities, and pH. Mortierellaceae and Cortinariaceae were positively associated with TP, pH, and DOC, Inocybaceae, Helvellaceae, and Thelephoraceae positioned in a direction similar to those of NAG, ACP and pH.

**Figure 6:**
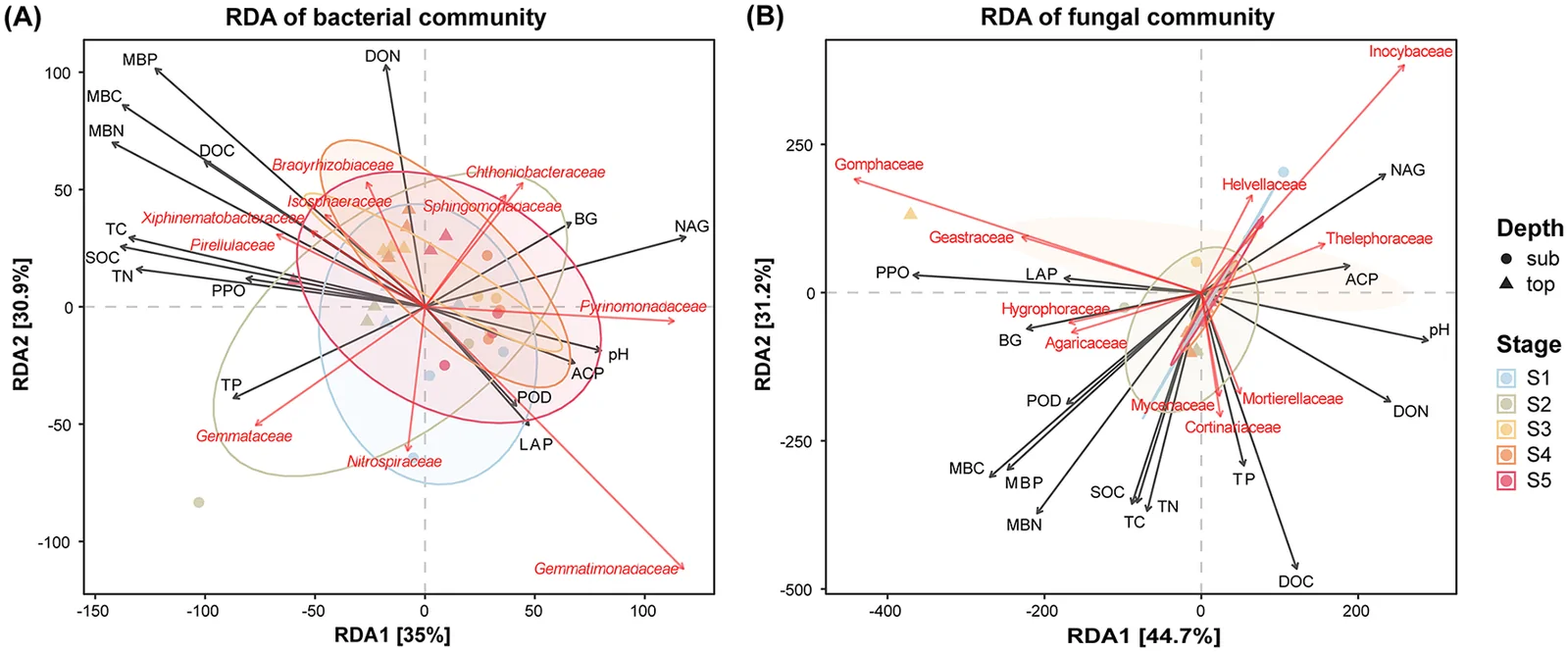
Redundancy analysis (RDA) illustrating the relationships between soil environmental variables and bacterial **(A)** and fungal **(B)** community composition. Black arrows denote environmental variables, whereas red arrows denote dominant family. Arrow direction indicates the direction of the association between environmental variables and community composition, while arrow length reflects the relative strength of their explanatory effects. TC, total carbon; TN, total nitrogen; TP, total phosphorus; SOC, soil organic carbon; DOC, dissolved organic carbon; DON, dissolved organic nitrogen; MBC, microbial biomass carbon; MBN, microbial biomass nitrogen; MBP, microbial biomass phosphorus; POD, peroxidase; PPO, polyphenol oxidase; BG, β-glucosidase; NAG, *N*-acetyl-β-D-glucosaminidase; ACP, acid phosphatase; LAP, leucine aminopeptidase.

Mantel tests further revealed contrasting relationships between bacterial and fungal communities and environmental variables (Figure 7). Bacterial community composition exhibited significant correlations with a broad range of environmental factors, including pH, TN, TC, TP, SOC, DOC, DON, MBC, MBN, MBP, BG, and NAG. In contrast, fungal community composition was significantly correlated only with pH and TP. Pearson correlation analysis showed that soil carbon, nitrogen, and phosphorus variables (TC, SOC, DOC, TN, and TP) were generally positively correlated with microbial biomass components (MBC, MBN, and MBP), whereas pH exhibited predominantly negative correlations with nutrient availability and microbial biomass variables. Collectively, these results demonstrate that bacterial communities were associated with a wider range of soil environmental factors, particularly nutrient availability, microbial biomass, and enzyme activities, whereas fungal communities exhibited stronger associations with soil pH and phosphorus availability, highlighting distinct environmental response patterns between bacterial and fungal communities during forest succession.

**Figure 7:**
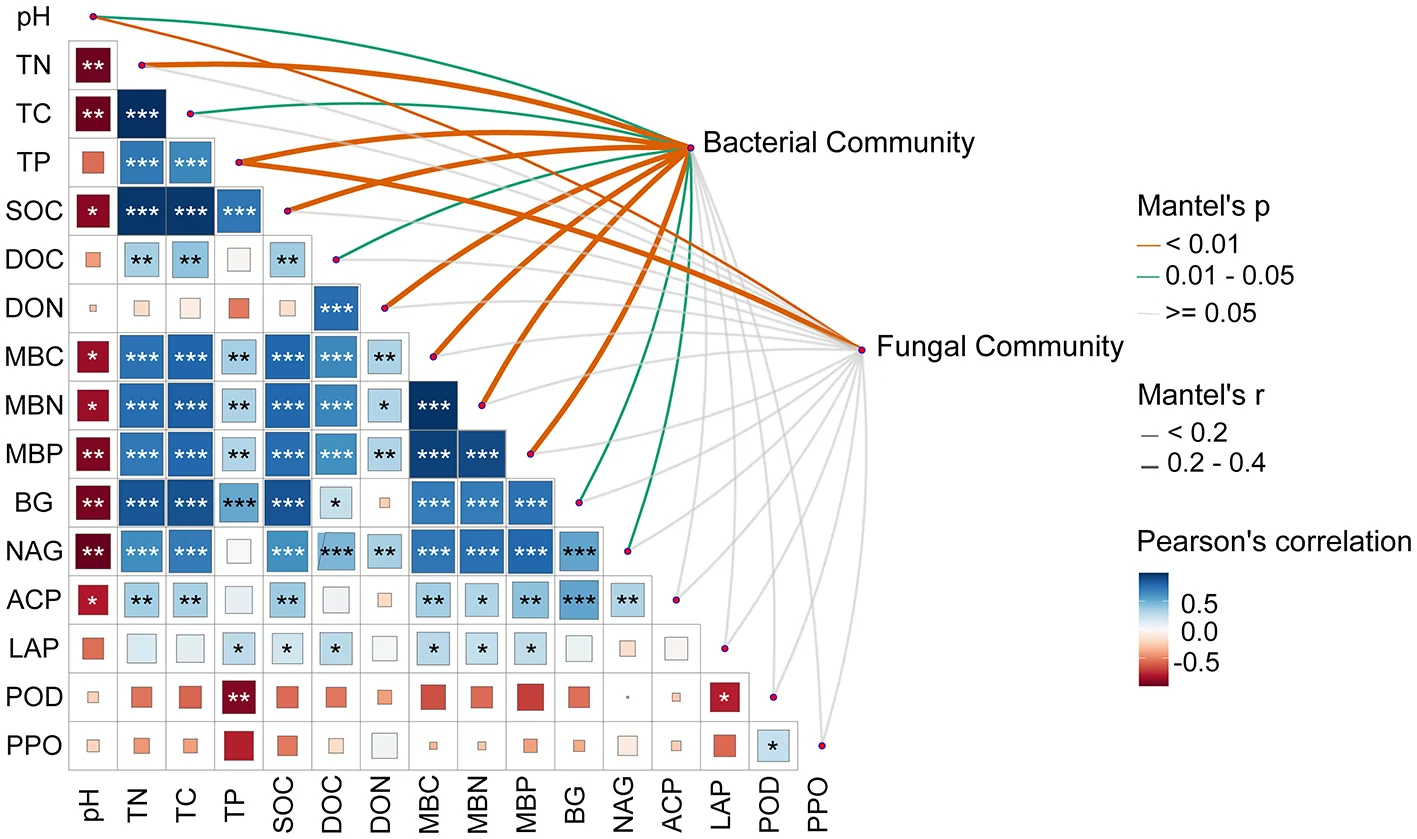
Mantel test analysis of the relationships between soil physicochemical properties, microbial biomass, enzyme activities, and bacterial and fungal community composition. The triangular matrix presents Pearson correlation coefficients among soil physicochemical properties, microbial biomass, and enzyme activities. Color indicates the direction and strength of the correlations, with blue and red representing positive and negative correlations, respectively; darker colors indicate larger absolute correlation coefficients. Asterisks denote the significance levels of Pearson correlations: * indicates *P* < 0.05, ** indicates *P* < 0.01, and *** indicates *P* < 0.001. Lines on the right represent Mantel test results linking the environmental-variable matrix to the bacterial and fungal community matrices. Bacterial and fungal community dissimilarities were calculated using Bray–Curtis distances, whereas Euclidean distances were used for the environmental-variable matrix. Line width represents the magnitude of the Mantel's *r* statistic, with thicker lines indicating stronger correlations. TC, total carbon; TN, total nitrogen; TP, total phosphorus; SOC, soil organic carbon; DOC, dissolved organic carbon; DON, dissolved organic nitrogen; MBC, microbial biomass carbon; MBN, microbial biomass nitrogen; MBP, microbial biomass phosphorus; POD, peroxidase; PPO, polyphenol oxidase; BG, β-glucosidase; NAG, *N*-acetyl-β-D-glucosaminidase; ACP, acid phosphatase; LAP, leucine aminopeptidase.

## 4 Discussion

### 4.1 Response patterns of soil bacterial and fungal communities during forest succession

Our results demonstrated that forest succession significantly reshaped soil bacterial and fungal community structure, while exerting no significant effects on microbial α-diversity (Figures 4, 5, Supplementary Table S4). PERMANOVA analysis showed that successional stage explained 26.7 and 28.9% of the variation in bacterial and fungal community structure, respectively, highlighting forest succession as a key driver of belowground microbial community reassembly. These findings support Hypothesis 1 that, under subalpine calcareous soil conditions, forest succession promotes microbial community reorganization through alterations in soil environmental conditions and resource availability. Notably, fungal communities exhibited slightly higher explanatory power than bacterial communities (28.9% vs. 26.7%), suggesting a relatively stronger successional sensitivity of fungi, which is consistent with Hypothesis 2. Fungi are more tightly coupled with plant-derived carbon inputs and nutrient acquisition, rendering their community assembly more responsive to shifts in vegetation composition and resource availability (Smith and Read, 2008; Phillips et al., 2013; Peay et al., 2016). Neither bacterial nor fungal richness, Shannon diversity, nor Pielou's evenness varied significantly along the successional gradient (Figure 4, Supplementary Table S4), indicating that forest succession did not simply lead to an overall increase or decline in microbial α-diversity, but instead primarily reshaped community composition. Previous studies have demonstrated that long-term environmental changes can restructure microbial communities primarily through dominant taxa replacement, niche differentiation, and changes in ecological interactions, without necessarily causing parallel shifts in α-diversity (Fierer et al., 2012; Shade et al., 2012; He et al., 2022; Wang et al., 2025). Therefore, the concurrent occurrence of pronounced microbial compositional turnover and stable α-diversity observed in this study suggests that microbial responses during subalpine forest succession were primarily driven by changes in community structure rather than variations in overall richness or evenness.

Bacterial communities exhibited stage-specific shifts in relative abundance of dominant taxa along the successional gradient. At the phylum level, the relative abundance of *Actinobacteriota* increased progressively in surface soils, whereas *Chloroflexi* declined during succession (Figure 2). At the family level, the relative abundance of *Nitrospiraceae* in the surfacesoil showed a continuous decrease, while *Micromonosporaceae* and *Pirellulaceae* showed increasing relative abundances across the successional stages in surface soil (Figure 3), indicating that bacterial taxa differed substantially in their ecological responses to successional changes in soil environments. *Nitrospiraceae*, as an important group involved in nitrification processes, is sensitive to variations in nitrogen availability and substrate supply. Its decline may reflect shifts in soil nitrogen cycling pathways associated with increasing plant nitrogen demand and changes in inorganic nitrogen availability during forest development. Specifically, enhanced plant nitrogen uptake and increasing immobilization of nitrogen into organic pools may reduce the availability of inorganic nitrogen substrates, thereby weakening the competitive advantage of nitrifying microorganisms (Prosser and Nicol, 2012; Kuypers et al., 2018). Although the relative abundances of dominant bacterial taxa varied across the forest succession gradient, bacterial α-diversity did not differ significantly among successional stages, indicating that changes in community structure were not accompanied by significant changes in α-diversity. This finding is consistent with Hypothesis 2 that bacterial communities primarily adapt to changes in soil resource availability and environmental conditions during forest succession through shifts in dominant taxa.

In contrast, fungal communities exhibited more pronounced successional reorganization, particularly in surface soils. Although Inocybaceae and Mortierellaceae occurred at relatively high abundances, their relative abundances did not differ significantly among successional stages. Several fungal taxa exhibited significant stage-specific differences, with distinct patterns of variation among taxa, indicating that the successional response of fungal communities was primarily characterized by stage-specific reorganization of individual taxa. Previous studies have shown that fungal community assembly is more tightly coupled with host plant composition, plant-derived carbon inputs, and soil nutrient availability than bacterial communities. Accordingly, fungal succession is jointly regulated by vegetation turnover, rhizosphere carbon supply, and changes in soil physicochemical properties (Phillips et al., 2013; Tedersoo et al., 2014; Peay et al., 2016; Hannula et al., 2017).

Overall, both bacterial and fungal communities exhibited stable α-diversity but continuous compositional shift throughout forest succession; however, they followed distinct ecological response pathways. Bacterial communities primarily exhibited stage-specific shifts in the relative abundances of dominant taxa, whereas fungal communities showed more pronounced changes in community composition reorganization. These contrasting patterns indicate that soil bacterial and fungal communities exhibited divergent successional responses to environmental changes during forest succession.

### 4.2 Environmental drivers shaping soil microbial community succession

The RDA and Mantel test results revealed that bacterial and fungal communities were regulated by distinct environmental drivers during forest succession (Figures 6, 7), providing further support for Hypothesis 2. During forest succession, surface soil pH increased from 7.15 at S1 to 7.49 at S5, whereas TP declined during the early successional stages (S1–S3) and subsequently remained relatively stable. In subsurface soil, TC, TN, and SOC exhibited a decreasing–increasing pattern, while DON reached a relatively high level at S4 (Table 2), suggesting that forest development was accompanied by stage-specific shifts in soil acidity–alkalinity and carbon, nitrogen, and phosphorus availability. Under the carbonate parent material background, the strong buffering capacity of soils may have alleviated the potential acidifying effects associated with coniferous litter inputs, thereby preventing a continuous decline in soil pH (Lapenis et al., 2004; Lucas et al., 2011; Wei et al., 2012). Therefore, the observed changes in soil environmental conditions may result from the combined influence of regional pedogenic background and vegetation succession.

Bacterial community composition exhibited significant associations with soil pH, SOC, TC, TN, TP, DOC, DON, microbial biomass (MBC, MBN, and MBP), and hydrolytic enzyme activities (BG and NAG) (Figure 7), indicating that bacterial communities responded broadly to variations in soil resource availability and environmental conditions. Previous studies have demonstrated that soil pH serves as a major environmental filter shaping bacterial community composition, while the availability of carbon, nitrogen, and phosphorus further influences bacterial resource utilization strategies and determines shifts in the relative abundance of specific taxa (Fierer and Jackson, 2006; Lauber et al., 2009; Rousk et al., 2010; Fierer, 2017). In the present study, *Pirellulaceae, Xiphinematobacteraceae*, and *Bradyrhizobiaceae* exhibited distribution patterns closely aligned with SOC, TC, TN, MBC, MBN, and MBP in the RDA ordination, *Nitrospiraceae* and *Gemmatimonadaceae* were closely associated with LAP and POD, whereas *Pyrinomonadaceae* showed a strong association with soil pH (Figure 6). These contrasting patterns suggest that different bacterial taxa exhibited distinct associations with and responses to changing soil conditions. Accordingly, changes in bacterial community structure during forest succession were accompanied by the shift of dominant taxa.

In contrast, fungal communities were primarily associated with soil pH and TP (Figure 7), suggesting that soil acidity–alkalinity conditions and phosphorus availability act as important environmental filters regulating fungal community assembly. The RDA results further showed that, Mortierellaceae and Cortinariaceae were closely associated with the soil pH, TP, and DOC, whereas Inocybaceae, Helvellaceae, and Thelephoraceae exhibited stronger relationships with pH, NAG, and ACP (Figure 6). These patterns indicate that fungal taxa differ substantially in their ecological strategies and responses to soil nutrient availability and microbial nutrient acquisition processes. Given that certain fungal groups, particularly ectomycorrhizal-associated taxa, their successional dynamics are likely shaped by the combined influences of vegetation composition, nutrient availability, and rhizosphere resource supply (Averill et al., 2014; Peay et al., 2016).

Overall, the changes in soil environmental conditions during forest succession were accompanied by shifts in microbial community composition, with bacterial and fungal communities exhibiting distinct environmental associations. Bacterial communities showed broader associations with multiple soil nutrients, dissolved organic matter fractions, microbial biomass, and enzyme activities, whereas fungal communities were more closely associated with soil pH and TP. These findings suggest that bacterial and fungal communities may respond to environmental changes through different pathways during forest succession.

### 4.3 Soil vertical heterogeneity regulates microbial response strategies during forest succession

In this study, both bacterial and fungal communities exhibited pronounced vertical differentiation across soil depths (Figures 2, 3).

In surface soils, bacterial community succession was primarily characterized by shifts in the relative abundances of dominant families. For instance, *Nitrosomonadaceae* and *Nitrospiraceae* generally declined along the successional gradient, whereas *Micromonosporaceae, Phyllobacteriaceae*, and *Pirellulaceae* increased at specific successional stages (Supplementary Table S2), suggesting that bacterial taxa exhibited distinct responses to forest development. Compared with bacteria, fungal communities showed distinct variation in the relative abundances of individual families across successional stages in surface soils, with families such as Atheliaceae, Hebelomataceae, Tuberaceae, and Hygrophoraceae showing distinct, and in some cases non-monotonic, patterns of abundance change (Supplementary Table S3). In subsurface soils, successional variations in both bacterial and fungal communities were relatively constrained, with significant differences detected for only a small number of families (Supplementary Tables S2, S3). These findings suggest that microbial responses to forest succession were depth-dependent, with more pronounced changes occurring in top soils than in subsurface soils.

This vertical differentiation may reflect differences in resource availability and plant-derived inputs between soil layers. Subsoils are directly influenced by litter deposition and root activity, resulting in greater inputs of plant-derived carbon and nutrients. Therefore, microbial communities in surface soils may respond more strongly to successional changes in vegetation composition and resource availability during forest development (Tedersoo et al., 2014; Peay et al., 2016). With increasing soil depth, the influence of aboveground plant inputs gradually declines, and slower resource turnover in subsurface soils may constrain microbial responses to successional changes. Meanwhile, root-derived inputs and soil mixing processes may partially buffer resource heterogeneity across soil layers, further reducing the magnitude of successional shifts in subsurface microbial communities (Fierer et al., 2003; Eilers et al., 2012; Jiao et al., 2018).

Additionally, the relatively broad sampling intervals used in this study (0–20 cm and 20–40 cm) may have masked finer-scale vertical heterogeneity, which could partly account for the weaker successional patterns detected in subsurface soils.

This study has several limitations. First, although the chronosequence approach is widely used to infer long-term successional patterns, it relies on the assumption that sites representing different successional stages have comparable initial environmental conditions and similar successional pathways, which is difficult to fully satisfy in natural ecosystems (Johnson and Miyanishi, 2008). Consequently, differences in historical legacies, microtopography, local microclimate, and initial soil conditions among sites may partially confound the observed patterns of microbial community succession. In this study, we minimized potential confounding effects by selecting sites within the same nature reserve with comparable slope aspects, parent materials, and soil types, and by integrating multiple lines of evidence from soil properties, enzyme activities, and microbial community characteristics. These approaches helped reduce biases associated with regional-scale environmental heterogeneity and anthropogenic disturbances, although fine-scale environmental variation and site-specific historical effects cannot be completely excluded. Future studies integrating long-term permanent plot monitoring, multi-scale observations, and manipulative experiments will be valuable for further disentangling microbial community dynamics and their environmental controls during forest succession.

## 5 Conclusions

Forest succession significantly altered soil microbial community structure rather than changes in α-diversity in subalpine forests of the Eastern Qilian Mountains. Bacterial and fungal communities exhibited divergent successional response strategies. Bacterial communities responded mainly through stage-specific shifts in the relative abundances of dominant taxa and were associated with a broad range of soil resource variables and environmental conditions, whereas fungal communities showed more pronounced community taxonomic reorganization and stronger associations with soil pH and TP. These findings highlight divergent successional trajectories and environmental response strategies of soil bacterial and fungal communities during subalpine forest development under calcareous soil conditions, providing new insights into microbial community assembly and their potential contributions to nutrient cycling and ecosystem functioning throughout forest succession.

## Data Availability

The original contributions presented in the study are included in the article/Supplementary material, further inquiries can be directed to the corresponding author.
