# Supplementary material for: Divergent successional responses and environmental drivers of soil bacterial and fungal communities in subalpine forests of the Eastern Qilian Mountains
Source: Front Microbiol. 2026 Oct 9;17:1968766. doi: 10.3389/fmicb.2026.1968766 (PMC13690955; doi:10.3389/fmicb.2026.1968766)
Supplement: Supplementary file 1 [file Table_1.docx]

**Supplementary Materials**

**Figure S1.** Relative abundances of soil bacterial (A, B) and fungal (C, D) phyla across different successional stages based on individual plot-level replicates. Each bar represents one independent plot-level replicate (n = 3 per successional stage). S1–S5 represent different successional stages，and “top” and “sub” denote topsoil (0–20 cm) and subsoil (20–40 cm), respectively.

**Table S1**. Meteorological and soil microclimatic conditions during the sampling period (16–17 July 2024).

| Time | Air temperature (6 m) (℃) | Relative humidity (6 m) (%) | Soil water content (20 cm) (cm^3^/cm^3^) | Soil temperature (20 cm) (℃) | Soil water content (40 cm) (cm^3^/cm^3^) | Soil temperature (40 cm) (℃) |
| --- | --- | --- | --- | --- | --- | --- |
| 2024/7/16 | 20.024 | 69.375 | 0.224 | 11.278 | 0.173 | 9.534 |
| 2024/7/17 | 20.680 | 63.840 | 0.216 | 11.481 | 0.170 | 9.721 |

Note: Environmental data were obtained from the continuous monitoring records of the Gansu Xinglong Mountain Forest Ecosystem Research Station and represent the environmental background of the study area during the sampling period rather than direct measurements from individual successional-stage plots. The original environmental data were recorded at 30-min intervals, and the values reported for each sampling day represent the mean of measurements recorded between 09:00 and 19:00 on that day.

**Table S2**. Relative abundances of the top 25 dominant bacterial family across different successional stages and soil depths.

| **Family** | **Depth** | **S1** | **S2** | **S3** | **S4** | **S5** | ***P* value** |
| --- | --- | --- | --- | --- | --- | --- | --- |
| *Haliangiaceae* | top | 0.55±0.10A | 0.38±0.17AB | 0.26±0.06B | 0.33±0.03AB | 0.36±0.10AB | 0.057 |
| *Haliangiaceae* | sub | 0.35±0.11a | 0.23±0.18a | 0.24±0.01a | 0.35±0.03a | 0.34±0.17a | 0.596 |
| *Nitrosomonadaceae* | top | 3.12±0.30A | 2.02±0.10B | 1.29±0.16BC | 0.95±0.14C | 1.36±0.75BC | <0.001 |
| *Nitrosomonadaceae* | sub | 2.25±0.43a | 1.53±0.89a | 1.54±0.29a | 1.38±0.17a | 1.70±0.22a | 0.270 |
| *Nitrospiraceae* | top | 2.59±0.23A | 2.40±0.21A | 1.11±0.41B | 0.89±0.27B | 1.09±0.33B | <0.001 |
| *Nitrospiraceae* | sub | 2.60±0.28a | 1.73±0.71ab | 1.22±0.36b | 1.07±0.29b | 1.41±0.30b | 0.009 |
| *Phycisphaeraceae* | top | 0.50±0.46A | 0.42±0.28A | 0.29±0.16A | 0.67±0.31A | 0.37±0.31A | 0.675 |
| *Phycisphaeraceae* | sub | 0.98±0.50a | 0.71±0.08a | 0.39±0.11a | 0.49±0.30a | 0.99±0.65a | 0.284 |
| *Pyrinomonadaceae* | top | 4.44±0.90A | 4.00±0.65A | 4.34±0.61A | 4.77±0.36A | 4.27±2.74A | 0.969 |
| *Pyrinomonadaceae* | sub | 4.93±1.38a | 3.76±2.72a | 7.20±1.85a | 6.08±1.60a | 6.25±1.93a | 0.306 |
| *Gemmatimonadaceae* | top | 2.34±0.62A | 2.67±0.31A | 3.01±0.64A | 2.68±0.21A | 2.74±0.87A | 0.736 |
| *Gemmatimonadaceae* | sub | 5.70±1.94a | 4.97±1.81a | 6.91±2.30a | 7.31±1.08a | 6.15±1.20a | 0.510 |
| *Gemmataceae* | top | 3.10±0.27A | 4.69±1.46A | 3.43±1.83A | 4.28±0.27A | 3.92±1.63A | 0.586 |
| *Gemmataceae* | sub | 3.56±1.95a | 4.65±2.02a | 2.42±0.27a | 2.42±1.20a | 3.24±0.93a | 0.347 |
| *Gaiellaceae* | top | 1.21±0.44A | 1.45±0.10A | 1.69±0.47A | 1.29±0.17A | 1.71±0.43A | 0.371 |
| *Gaiellaceae* | sub | 1.12±0.18a | 1.83±0.52a | 1.53±0.23a | 1.36±0.23a | 1.44±0.29a | 0.159 |
| *Solirubrobacteraceae* | top | 0.27±0.08A | 0.37±0.03A | 0.59±0.14A | 0.58±0.09A | 1.45±1.60A | 0.348 |
| *Solirubrobacteraceae* | sub | 0.12±0.10a | 0.89±0.94a | 0.29±0.08a | 0.46±0.09a | 0.35±0.15a | 0.319 |
| *Micromonosporaceae* | top | 0.58±0.10B | 0.49±0.23B | 1.14±0.40AB | 0.84±0.24AB | 1.50±0.34A | 0.007 |
| *Micromonosporaceae* | sub | 0.28±0.04b | 0.80±0.26ab | 0.48±0.07ab | 0.80±0.21ab | 0.84±0.30a | 0.025 |
| *Mycobacteriaceae* | top | 0.29±0.08A | 0.60±0.06A | 1.31±0.21A | 0.96±0.11A | 1.36±1.42A | 0.270 |
| *Mycobacteriaceae* | sub | 0.11±0.04a | 1.04±0.94a | 0.43±0.09a | 0.65±0.23a | 0.51±0.23a | 0.224 |
| *Nocardioidaceae* | top | 0.26±0.14A | 0.44±0.01A | 0.87±0.13A | 0.83±0.06A | 1.05±1.02A | 0.274 |
| *Nocardioidaceae* | sub | 0.17±0.10a | 0.54±0.47a | 0.26±0.06a | 0.52±0.09a | 0.39±0.21a | 0.322 |
| *Vicinamibacteraceae* | top | 3.18±0.03A | 2.82±0.12A | 2.92±0.81A | 3.54±0.52A | 3.82±1.66A | 0.602 |
| *Vicinamibacteraceae* | sub | 2.81±0.25a | 2.47±1.22a | 2.70±0.38a | 2.77±0.11a | 3.70±0.12a | 0.182 |
| *Hyphomicrobiaceae* | top | 1.96±0.44A | 1.66±0.25A | 1.86±0.53A | 1.64±0.25A | 1.85±0.79A | 0.910 |
| *Hyphomicrobiaceae* | sub | 1.27±0.35a | 1.32±0.64a | 1.80±0.23a | 1.75±0.30a | 1.76±0.29a | 0.321 |
| *Chthoniobacteraceae* | top | 2.55±0.23AB | 3.07±0.37AB | 4.23±0.76A | 3.35±1.64AB | 1.79±0.63B | 0.060 |
| *Chthoniobacteraceae* | sub | 1.83±1.39a | 1.46±0.92a | 5.25±2.97a | 4.44±1.37a | 2.25±1.26a | 0.082 |
| *Sphingomonadaceae* | top | 0.92±0.04A | 0.95±0.22A | 1.48±0.16A | 1.76±0.43A | 1.21±0.85A | 0.175 |
| *Sphingomonadaceae* | sub | 0.62±0.46a | 0.75±0.63a | 1.47±0.20a | 1.68±0.57a | 1.11±0.41a | 0.092 |
| *Phyllobacteriaceae* | top | 0.37±0.05B | 0.44±0.07AB | 0.72±0.14AB | 0.88±0.33A | 0.46±0.12AB | 0.021 |
| *Phyllobacteriaceae* | sub | 0.16±0.12b | 0.24±0.11b | 0.37±0.03ab | 0.59±0.16a | 0.30±0.07b | 0.006 |
| *Xiphinematobacteraceae* | top | 0.89±0.54A | 1.63±0.48A | 1.71±0.51A | 1.49±0.77A | 0.99±0.91A | 0.467 |
| *Xiphinematobacteraceae* | sub | 0.41±0.09b | 0.71±0.25ab | 0.64±0.27ab | 1.10±0.36a | 0.42±0.21ab | 0.044 |
| *Methyloligellaceae* | top | 0.69±0.23A | 0.83±0.18A | 0.81±0.30A | 0.71±0.07A | 0.60±0.19A | 0.667 |
| *Methyloligellaceae* | sub | 0.39±0.11a | 0.54±0.01a | 0.46±0.17a | 0.52±0.12a | 0.33±0.15a | 0.270 |
| *Xanthobacteraceae* | top | 2.00±0.18A | 1.91±0.16A | 2.51±0.15A | 2.14±0.40A | 2.39±0.40A | 0.110 |
| *Xanthobacteraceae* | sub | 0.96±0.20b | 1.29±0.38ab | 1.68±0.24a | 1.66±0.08a | 1.46±0.20ab | 0.024 |
| *Bradyrhizobiaceae* | top | 0.69±0.11A | 0.89±0.07A | 2.01±0.92A | 1.42±0.74A | 1.07±0.41A | 0.104 |
| *Bradyrhizobiaceae* | sub | 0.25±0.04b | 0.52±0.21ab | 0.74±0.15a | 0.72±0.16a | 0.49±0.13ab | 0.015 |
| *Pirellulaceae* | top | 3.55±0.16B | 5.16±0.59AB | 4.24±1.12B | 6.18±0.58A | 4.12±0.72B | 0.008 |
| *Pirellulaceae* | sub | 3.02±1.09a | 2.83±0.20a | 2.30±0.28a | 3.46±0.55a | 3.73±0.58a | 0.120 |
| *Isosphaeraceae* | top | 0.26±0.04A | 0.62±0.13A | 1.46±1.34A | 2.09±0.17A | 1.49±1.21A | 0.117 |
| *Isosphaeraceae* | sub | 0.27±0.30a | 0.91±0.67a | 0.44±0.21a | 0.75±0.37a | 0.66±0.25a | 0.357 |
| *Pseudonocardiaceae* | top | 0.41±0.23A | 0.67±0.15A | 0.74±0.22A | 0.72±0.15A | 0.90±0.39A | 0.253 |
| *Pseudonocardiaceae* | sub | 0.20±0.07a | 0.47±0.24a | 0.40±0.31a | 0.60±0.25a | 0.41±0.36a | 0.521 |
| *Others* | top | 51.18±0.77A | 48.08±2.07AB | 43.90±3.11BC | 41.78±3.36C | 45.23±0.49ABC | 0.004 |
| *Others* | sub | 56.14±0.63a | 55.05±4.09ab | 50.32±0.87bc | 46.81±1.47c | 50.09±0.24bc | 0.001 |

Note: Data are presented as mean ± standard deviation (mean ± SD). S1–S5 represent different successional stages, and top and sub indicate surface and subsurface soils, respectively. *P* values indicate the significance of differences in bacterial genus relative abundances among different successional stages.

**Table S3**. Relative abundances of the top 25 dominant fungal family across different successional stages and soil depths.

| **Family** | **Depth** | **S1** | **S2** | **S3** | **S4** | **S5** | **P_value** |
| --- | --- | --- | --- | --- | --- | --- | --- |
| Inocybaceae | top | 23.36±16.29A | 10.98±3.63A | 31.42±24.70A | 16.28±7.54A | 28.15±6.62A | 0.420 |
| Inocybaceae | sub | 42.11±27.94a | 26.52±19.70a | 39.09±8.79a | 27.84±7.36a | 40.41±23.09a | 0.771 |
| Atheliaceae | top | 0.00±0.00B | 0.07±0.03B | 0.21±0.15B | 0.24±0.11B | 0.59±0.09A | <0.001 |
| Atheliaceae | sub | 0.00±0.00a | 0.14±0.21a | 1.20±0.68a | 0.74±0.59a | 0.39±0.40a | 0.049 |
| Cortinariaceae | top | 0.81±0.26A | 26.98±21.92A | 5.15±6.46A | 13.99±20.15A | 0.93±0.80A | 0.172 |
| Cortinariaceae | sub | 0.21±0.36a | 2.89±4.09a | 1.97±1.65a | 2.60±2.18a | 4.67±5.93a | 0.636 |
| Clavulinaceae | top | 0.82±0.90A | 1.86±1.52A | 0.05±0.05A | 0.19±0.29A | 0.14±0.06A | 0.091 |
| Clavulinaceae | sub | 1.18±1.35a | 0.44±0.38a | 1.61±1.39a | 1.99±2.47a | 0.12±0.21a | 0.494 |
| Clavariaceae | top | 0.66±0.11AB | 1.23±0.74A | 0.29±0.22AB | 0.68±0.41AB | 0.10±0.04B | 0.045 |
| Clavariaceae | sub | 0.27±0.30a | 1.31±1.05a | 2.13±1.21a | 0.91±0.79a | 0.17±0.27a | 0.079 |
| Hygrophoraceae | top | 0.39±0.58B | 5.63±0.80A | 0.14±0.06B | 0.69±1.13B | 0.01±0.01B | <0.001 |
| Hygrophoraceae | sub | 0.27±0.46a | 5.33±6.72a | 0.39±0.14a | 0.04±0.05a | 0.17±0.16a | 0.219 |
| Geastraceae | top | 2.06±1.84A | 0.19±0.16A | 0.03±0.05A | 0.00±0.01A | 1.00±1.13A | 0.111 |
| Geastraceae | sub | 1.73±2.74a | 21.44±36.61a | 0.60±0.99a | 0.02±0.03a | 0.22±0.12a | 0.467 |
| Tremellaceae | top | 0.57±0.53A | 1.85±1.91A | 2.56±3.54A | 0.64±0.46A | 1.07±0.78A | 0.651 |
| Tremellaceae | sub | 1.64±2.28a | 6.86±9.08a | 0.52±0.32a | 0.39±0.33a | 0.48±0.36a | 0.330 |
| Gomphaceae | top | 0.00±0.00A | 0.15±0.06A | 30.65±52.53A | 0.51±0.36A | 0.20±0.06A | 0.448 |
| Gomphaceae | sub | 0.00±0.00a | 1.19±2.01a | 3.95±6.73a | 0.08±0.02a | 0.04±0.05a | 0.513 |
| Umbelopsidaceae | top | 0.31±0.29A | 2.53±3.30A | 2.73±2.94A | 0.16±0.15A | 0.27±0.16A | 0.331 |
| Umbelopsidaceae | sub | 0.31±0.54a | 2.01±1.65a | 0.74±0.75a | 0.24±0.32a | 0.06±0.05a | 0.108 |
| Trichocomaceae | top | 0.14±0.23A | 0.60±0.09A | 0.97±1.50A | 0.48±0.23A | 0.25±0.15A | 0.633 |
| Trichocomaceae | sub | 0.09±0.07a | 0.66±1.08a | 0.07±0.09a | 0.04±0.03a | 0.08±0.13a | 0.502 |
| Hebelomataceae | top | 2.02±0.86A | 0.95±0.30AB | 0.11±0.10B | 0.03±0.03B | 0.11±0.09B | <0.001 |
| Hebelomataceae | sub | 1.09±0.66a | 0.40±0.29ab | 0.30±0.35ab | 0.02±0.02b | 0.17±0.17ab | 0.039 |
| Tuberaceae | top | 0.99±0.01A | 0.08±0.09B | 0.14±0.17B | 0.16±0.12B | 0.01±0.01B | <0.001 |
| Tuberaceae | sub | 0.96±0.92a | 0.11±0.15a | 0.42±0.61a | 0.30±0.41a | 0.18±0.26a | 0.386 |
| Pyronemataceae | top | 0.82±0.62A | 0.90±0.22A | 0.43±0.38A | 0.52±0.59A | 1.14±0.79A | 0.545 |
| Pyronemataceae | sub | 1.96±1.16a | 0.57±0.23a | 0.48±0.30a | 1.46±1.57a | 0.80±0.49a | 0.290 |
| Helvellaceae | top | 1.11±0.90A | 0.35±0.21A | 0.22±0.28A | 0.17±0.13A | 0.73±0.12A | 0.111 |
| Helvellaceae | sub | 2.79±1.88a | 0.57±0.28ab | 0.49±0.24ab | 0.08±0.10b | 0.72±0.65ab | 0.030 |
| Mycenaceae | top | 6.78±9.84A | 0.66±0.57A | 0.39±0.29A | 1.65±1.45A | 1.13±0.09A | 0.426 |
| Mycenaceae | sub | 0.25±0.24a | 0.06±0.06a | 0.06±0.09a | 0.10±0.17a | 2.29±3.03a | 0.268 |
| Microascaceae | top | 1.11±0.76A | 0.60±0.56A | 0.64±0.92A | 0.54±0.61A | 0.63±0.18A | 0.828 |
| Microascaceae | sub | 0.31±0.49a | 0.23±0.29a | 0.14±0.21a | 0.13±0.21a | 0.08±0.13a | 0.884 |
| Mortierellaceae | top | 10.24±5.12A | 4.10±1.95A | 2.68±2.77A | 8.23±1.29A | 8.79±3.58A | 0.071 |
| Mortierellaceae | sub | 5.07±5.26a | 1.93±1.13a | 1.82±0.61a | 3.84±2.46a | 3.32±2.68a | 0.636 |
| Herpotrichiellaceae | top | 4.04±2.96A | 2.38±0.31A | 2.04±1.21A | 4.72±0.34A | 1.94±0.40A | 0.131 |
| Herpotrichiellaceae | sub | 0.64±0.61a | 0.44±0.25a | 0.50±0.32a | 1.66±1.15a | 0.42±0.23a | 0.150 |
| Pezizaceae | top | 0.69±0.62AB | 0.05±0.02B | 0.01±0.01B | 0.00±0.00B | 1.06±0.21A | 0.003 |
| Pezizaceae | sub | 0.63±0.34a | 0.05±0.05a | 0.05±0.06a | 0.01±0.01a | 1.06±1.06a | 0.100 |
| Nectriaceae | top | 2.30±1.42B | 2.32±0.06B | 0.94±0.84B | 2.69±0.80B | 5.83±0.70A | <0.001 |
| Nectriaceae | sub | 2.28±3.21a | 1.28±1.18a | 0.53±0.25a | 1.16±0.95a | 1.37±0.23a | 0.759 |
| Clavicipitaceae | top | 0.44±0.32A | 0.06±0.05A | 0.05±0.04A | 0.27±0.41A | 2.28±1.81A | 0.041 |
| Clavicipitaceae | sub | 0.27±0.46a | 0.02±0.02a | 0.03±0.05a | 0.02±0.02a | 0.31±0.50a | 0.622 |
| Thelephoraceae | top | 2.99±1.05A | 3.07±1.56A | 1.27±0.94A | 6.46±5.16A | 5.04±2.39A | 0.237 |
| Thelephoraceae | sub | 2.84±0.56a | 2.27±1.65a | 2.67±1.08a | 6.94±3.23a | 5.48±3.28a | 0.110 |
| Agaricaceae | top | 0.73±0.61A | 0.06±0.03A | 0.31±0.36A | 10.79±18.19A | 2.16±2.24A | 0.491 |
| Agaricaceae | sub | 1.52±1.43a | 0.11±0.16a | 0.08±0.02a | 0.22±0.19a | 0.83±0.49a | 0.113 |
| Others | top | 24.72±5.52A | 26.87±13.44A | 14.13±11.36A | 23.31±14.31A | 29.53±6.75A | 0.515 |
| Others | sub | 20.92±11.91a | 14.89±5.12a | 35.74±7.45a | 45.55±15.84a | 32.78±22.58a | 0.135 |

Note: Data are presented as mean ± standard deviation (mean ± SD). S1–S5 represent different successional stages, and top and sub indicate surface and subsurface soils, respectively. *P* values indicate the significance of differences in fungal genus relative abundances among different successional stages.

**Table S4 Effects of forest succession stage, soil depth, and their interaction on soil physicochemical properties, microbial biomass, and α-diversity based on linear mixed-effects models.**

|  | Indicators | Succession stage | Depth | Succession stage×Depth |
| --- | --- | --- | --- | --- |
| Bacteria | Richness | 0.504 | 0.791 | 0.834 |
|  | Shannon | 0.843 | 0.878 | 0.980 |
|  | Pielou | 0.675 | 0.607 | 0.948 |
| Fungi | Richness | 0.975 | 0.085 | 0.876 |
|  | Shannon | 0.951 | 0.440 | 0.970 |
|  | Pielou | 0.895 | 0.670 | 0.981 |

Note: The numerical values in the table represent *P* values.
